# A multi-targeted, barrier-penetrating inhalable nanoplatform for simultaneous immunomodulation and antifibrotic therapy in idiopathic pulmonary fibrosis

**DOI:** 10.1016/j.mtbio.2026.103805

**Published:** 2026-11-06

**Authors:** Yuyao Sun, Chenggong Yu, Chaochao Zhang, Ying Li, Wenqing Zhang, Bowei Li, Mengli Song, Lin Wang, Bowen Li, Qingwen Xu

**Affiliations:** a Department of Pharmacy, The First Affiliated Hospital of Wannan Medical University (Yijishan Hospital of Wannan Medical University), Wuhu, 241001, China; b School of Pharmacy, Wannan Medical University, Wuhu, 241002, China; c School of Pharmacy, Anhui Medical University, Hefei, 230032, China; d Anhui Innovative Center for Drug Basic Research of Metabolic Diseases, Wannan Medical University, Wuhu, 241002, China

**Keywords:** Idiopathic pulmonary fibrosis, Inhalable nanoparticles, Collagenase, Barrier penetration, Inflammation modulation

## Abstract

Idiopathic pulmonary fibrosis (IPF) is a progressive and fatal lung disorder characterized by excessive extracellular matrix (ECM) accumulation, myofibroblast activation, and chronic inflammation. Current pharmacotherapies are hindered by modest anti-fibrotic efficacy and poor drug accumulation within fibrotic lesions. Herein, we developed an inhalable, collagenase-modified liposomal nanoplatform co-encapsulating methylprednisolone and pirfenidone (termed CMPNPs) to address these limitations. The CMPNPs exhibited favorable physicochemical properties and retained collagenase activity under atomization-induced shear forces, enabling efficient penetration through collagen-dense barriers and enhanced lung accumulation. In vitro, CMPNPs suppressed TGF-β1-induced myofibroblast differentiation and migration, and shifted macrophage polarization away from pro-fibrotic and pro-inflammatory phenotypes. In a bleomycin-induced IPF mouse model, inhaled CMPNPs improved survival, reduced collagen deposition and fibrotic lesions, and demonstrated superior lung retention and anti-fibrotic efficacy compared to intravenous administration. Collectively, this multi-targeted, barrier-penetrating strategy overcomes key bottlenecks of current IPF therapies and offers a promising translatable approach for treating progressive pulmonary fibrosis.

## Highlights

• Methylprednisolone and pirfenidone are co-loaded in an inhalable liposome for IPF.
• Surface-anchored collagenase digests the dense ECM barrier for deep lung penetration.
• The dual-drug nanomedicine suppresses both M1 and M2 macrophage polarization.
• The nanoplatform inhibits myofibroblast activation and migration in fibrotic lungs.
• Inhalation outperforms intravenous delivery, improving survival in IPF model mice.

## Graphical abstract

Inhaled CPMNPs penetrate the collagen barrier in fibrotic lungs and concurrently modulate immune responses and suppress fibroblast activation, halting idiopathic pulmonary fibrosis progression.

**Figure:**
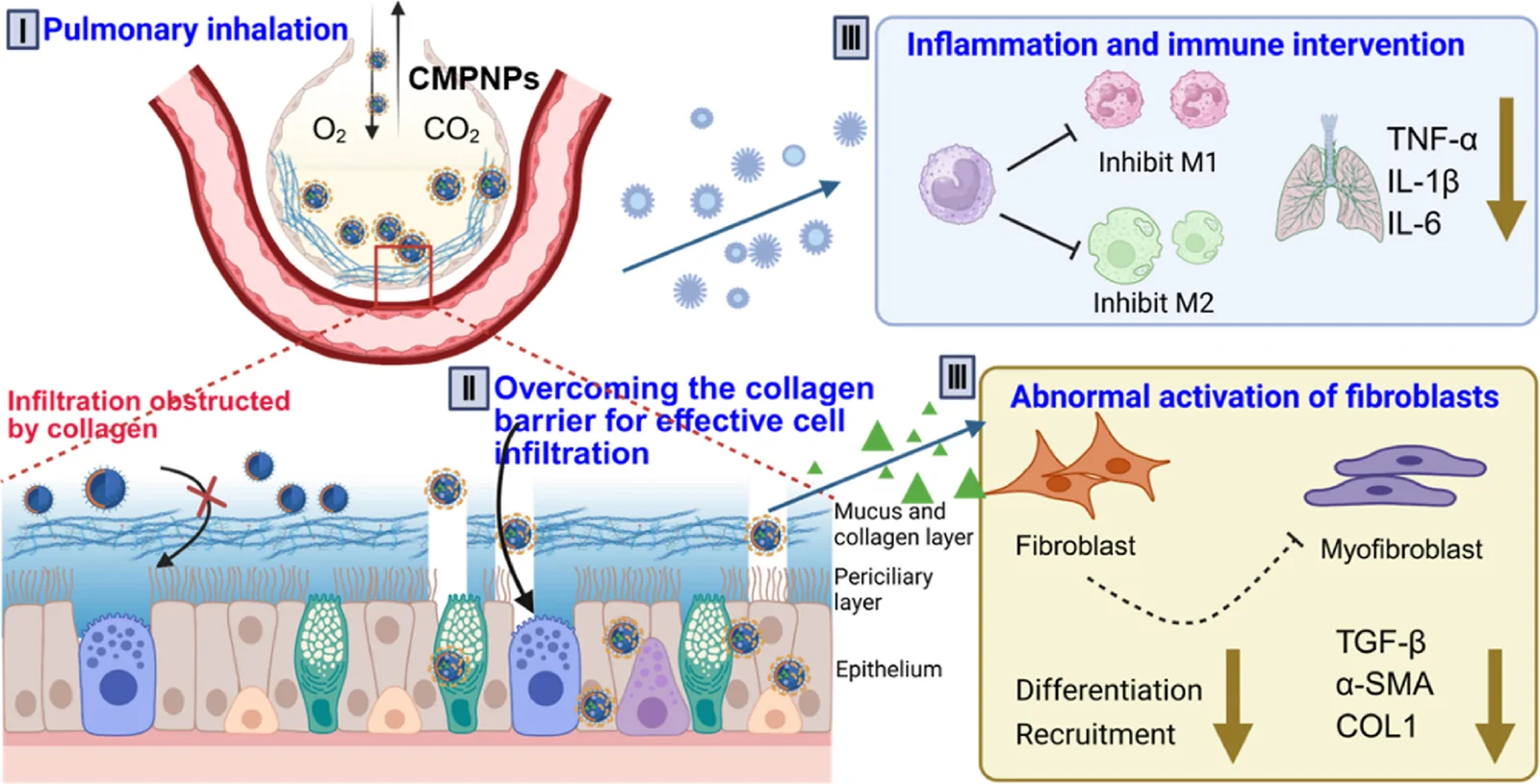

## 1 Introduction

Idiopathic pulmonary fibrosis (IPF) is a chronic and progressive interstitial lung disease in which sustained alveolar injury, dysregulated immune responses, and persistent fibroblast activation culminate in irreversible scarring and respiratory failure [1]. With a median survival of only 2–4 years after diagnosis and a 5-year mortality rate exceeding 60%, its prognosis is worse than that of many malignancies [2]. Pirfenidone (PFD), nintedanib, and the recently approved phosphodiesterase 4B inhibitor nerandomilast are currently the only approved drugs for IPF [3], [4]. Although these agents represent important advances, their benefit is largely limited to slowing lung-function decline in mild-to-moderate disease. None of them can halt or reverse established fibrosis, and their use is frequently complicated by gastrointestinal and hepatic tolerability issues, which underscores the persistent gap in effective treatment. Beyond these small molecules, emerging strategies such as mesenchymal stem cell-based therapies have shown potential in modulating fibrotic processes [5], [6], [7], [8]. However, their clinical translation is hindered by challenges in standardization, in vivo stability, and mechanistic understanding. Despite these research advances, the overall therapeutic landscape for IPF remains inadequate, and novel strategies capable of arresting or even reversing pulmonary fibrosis are urgently needed.

The pathogenesis of IPF involves a complex interplay of persistent inflammation, immune dysregulation, and aberrant fibroblast activation [9], [10]. Macrophages play a central role in regulating pulmonary immune homeostasis. During the early injury phase, pro-inflammatory M1 macrophages become activated and release cytokines such as IL-6, TNF-α, and TGF-β, thereby exacerbating tissue damage [11], [12]. As fibrosis progresses, a shift toward anti-inflammatory M2 macrophages occurs. These cells may suppress inflammation but concurrently promote collagen deposition and fibrotic remodeling [13]. Consequently, restoring the balance between M1 and M2 polarization has emerged as a promising therapeutic strategy [14], [15], [16]. Simultaneously, activated fibroblasts differentiate into myofibroblasts under the influence of transforming growth factor -β (TGF-β) and other mediators, leading to excessive synthesis and deposition of extracellular matrix (ECM) components, which ultimately results in lung stiffening and architectural distortion [17], [18]. Although targeting signaling pathways such as TGF-β/Smad or JAK/STAT can modulate both inflammatory and fibrotic processes [19], [20], [21], monotherapies often fall short due to the redundant and interconnected nature of IPF pathogenesis. Accordingly, a combinatorial strategy that simultaneously addresses immune imbalance and fibroblast activation represents a rational and potentially more effective approach [22].

Inhalation delivery provides a direct route to the lungs, maximizing local drug concentration while minimizing systemic exposure [23], [24]. Recent advances in inhalable nanoplatforms have highlighted the potential of smart nanocarriers to achieve sequential drug release and remodel pulmonary immune homeostasis [25], [26], [27]. However, effective drug delivery in IPF is severely hindered by the dense, collagen-rich ECM, which acts as a physical barrier that limits penetration into fibrotic areas and reduces drug bioavailability [28]. Although cationic carriers or cell-penetrating peptides have been employed to enhance tissue retention [29], [30], [31], they do not actively degrade the pathological collagen barrier. Collagenase (ColE), a natural enzyme responsible for collagen degradation, offers a compelling solution to this challenge [32], [33], [34]. By enzymatically cleaving collagen fibers, ColE can enhance nanoparticle penetration, improve drug access to affected cells, and potentially broaden the therapeutic window for IPF treatment.

Herein, we designed an inhalable, collagenase-modified liposomal nanoplatform co-loaded with methylprednisolone (MPS) and pirfenidone (PFD), termed CMPNPs, to overcome the collagen barrier and synergistically target both immune dysregulation and fibroblast activation in IPF (Scheme 1). The surface-conjugated ColE facilitates degradation of the collagen-rich ECM, thereby promoting deep tissue penetration. MPS modulates inflammatory response and macrophage polarization, while PFD directly inhibits fibroblast proliferation, migration, and differentiation into myofibroblasts. We systematically characterized the physicochemical properties, drug release profiles, and collagenase activity of CMPNPs, and evaluated their ability to suppress fibroblast activation and modulate macrophage polarization in vitro. Furthermore, in a bleomycin-induced murine model of IPF, inhalational administration of CMPNPs significantly attenuated lung fibrosis, reduced collagen deposition, improved lung function, and enhanced survival. Collectively, these findings demonstrate the potential of this multi-targeted, barrier-penetrating nanotherapeutic strategy for the treatment of progressive pulmonary fibrosis.

**Scheme 1:**
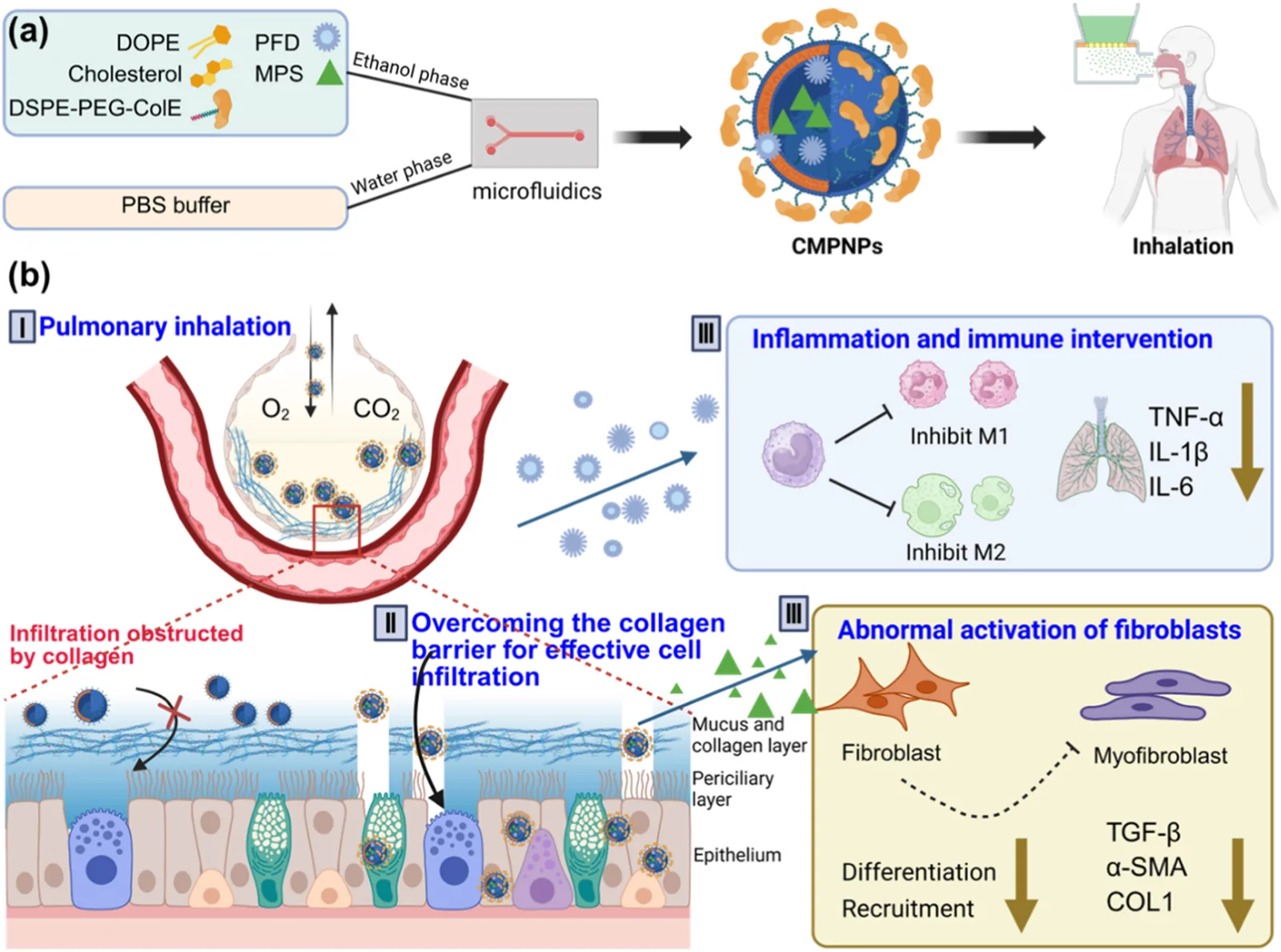
Schematic illustration of the fabrication and therapeutic mechanism of CMPNPs for IPF. (a) CMPNPs are prepared via microfluidics and subsequently administered through pulmonary inhalation. (b) Upon pulmonary inhalation, CMPNPs first penetrate the pathological collagen barrier, enabling efficient infiltration into fibrotic lesions. They then exert dual therapeutic effects: on one hand, modulating inflammatory and immune responses to alleviate the pro-fibrotic microenvironment; on the other hand, directly suppressing abnormal fibroblast activation, thereby halting the progression of IPF.

## 2 Experimental section

### 2.1 Materials

The following chemicals were used as received: 1,2-dioleoyl-sn-glycero-3-phosphoethanolamine (DOPE, Macklin), cholesterol (Energy Chemical), DSPE-PEG(2000)-COOH (Chongqing Yusi Pharmaceutical Technology), pirfenidone (PFD, Macklin), methylprednisolone (MPS, Heowns), 1-(3-dimethylaminopropyl)-3-ethylcarbodiimide hydrochloride (EDC, Energy Chemical), N-hydroxysuccinimide (NHS, Energy Chemical), 4-morpholineethanesulfonic acid (MES, Aladdin), and type I collagenase (ColE, Macklin). DMEM/F-12 (1:1) medium and fetal bovine serum (FBS) were purchased from Gibco. Trypsin and penicillin-streptomycin were obtained from NCM Biotech. Total RNA was extracted using the FastPure Cell/Tissue Total RNA Isolation Kit V2 (Vazyme). First-strand cDNA was synthesized with the HiScript II 1st Strand cDNA Synthesis Kit (+gDNA wiper, Vazyme), and quantitative PCR (qPCR) was performed using ChamQ SYBR qPCR Master Mix (Vazyme). ELISA kits, including the Mouse IL-6 and TNF-α ELISA kits, were purchased from MULTI SCIENCES (Lianke, Zhejiang, China). All qPCR primers (sequences listed in Table S1) were synthesized by Tsingke Biotechnology Co., Ltd.

The antibodies used in this work are described as follows: anti-COL1A1 rabbit pAb (1:1000 for Western Blotting, ABclonal, China, Catalog: A16699), anti-α-SMA rabbit mAb (1:5000 for Western Blotting, ABclonal, China, Catalog: A17910), anti-CD206 rabbit mAb (1:1000 for Western Blotting, Proteintech, China, Catalog: 18704-1-AP), anti-IL-10 rabbit mAb (1:2000 for Western Blotting, Proteintech, China, Catalog: 60269-1-Ig), anti-CD86 rabbit mAb (1:3000 for Western Blotting, Proteintech, China, Catalog: 83213-6-RR), anti-GAPDH antibody (1:1000 for Western Blotting, Biosharp, China), HRP-conjugated Affinipure Goat anti-rabbit IgG(H + L) (1:10000 for Western Blotting, Bioworld, USA, Catalog:BS13278), HRP-conjugated Affinipure Goat anti-mouse IgG(H + L) (1:10000 for Western Blotting, Bioworld, USA, Catalog: BS12478).

### 2.2 Synthesis of DSPE-PEG-ColE

DSPE-PEG-ColE was synthesized via a carbodiimide (EDC/NHS) coupling reaction. Briefly, DSPE-PEG-COOH (20 mg), NHS (46 mg), and EDC (31 mg) were dissolved in anhydrous dimethyl sulfoxide (DMSO, 5 mL) and activated at room temperature for 30 min. Separately, collagenase (ColE, 40 mg) was dissolved in MES buffer (0.1 M, 10 mL, pH 6.0). The ColE solution was then added dropwise to the activated mixture. The reaction proceeded overnight at room temperature under gentle agitation. The crude product was purified by dialysis (MWCO: 1000 Da) against deionized water for 48 h, with water changed every 8 h. The final conjugate was obtained as a light brown solid after lyophilization.

### 2.3 Nanoparticle preparation

Drug-loaded liposomes were prepared using a microfluidic-assisted ethanol injection method. Briefly, a lipid stock solution was prepared by dissolving DOPE (5 mg), cholesterol (10 mg), and DSPE-PEG-COOH (2 mg) in anhydrous ethanol (5 mL) to a final concentration of 3.4 mg/mL. To prepare MPNPs, pirfenidone (PFD, 5 mg) and methylprednisolone (MPS, 5 mg) were dissolved in 2 mL of this lipid solution to form the ethanol phase. This phase and an aqueous phase (5 mL PBS) were loaded into separate syringes and rapidly mixed via a microfluidic chip. The mixture was immediately dialyzed (MWCO: 3.5 kDa) against PBS to remove ethanol and promote liposome formation, followed by concentration using a 3 kDa MWCO ultrafiltration tube. Blank nanoparticles (BNPs) and single drug-loaded nanoparticles (MNPs, PNPs) were prepared analogously by omitting or adjusting drug amounts. For CMPNPs, DSPE-PEG-COOH in the lipid stock was replaced with an equimolar amount of the pre-synthesized DSPE-PEG-ColE conjugate.

### 2.4 Nanoparticle characterization

The hydrodynamic diameter and zeta potential of the nanoparticles were measured by dynamic light scattering (DLS) using a Litesizer 500 particle analyzer (Anton Paar). UV-vis absorption spectra were acquired with an Ultrospec 7000 spectrophotometer (Biochrom), and Fourier-transform infrared (FTIR) spectra were recorded on a Nicolet IS5 spectrometer (Thermo Fisher Scientific). For morphological assessment, a droplet of the liposome dispersion was deposited onto a carbon-coated copper grid, air-dried, and imaged using a Hitachi HT7800 transmission electron microscope (TEM) operating at an acceleration voltage of 100 kV. This work was partially carried out at WanNan Medical College Electron Microscopy Center.

### 2.5 Loading efficiency (LE) and encapsulation efficiency (EE)

Drug loading and encapsulation parameters were derived from HPLC measurements. Separations were carried out on an XBridge™ Shield RP18 column (4.6 × 250 mm, 5 μm) maintained at 30°C, with a flow rate of 1.0 mL/min and a 10 μL injection volume. For pirfenidone (PFD), detection was at 310 nm using an isocratic mobile phase of water/acetonitrile (70:30, v/v). For methylprednisolone (MPS), detection was at 247 nm with a mobile phase of water/acetonitrile (80:20, v/v). Individual standard curves for PFD and MPS were generated from methanolic solutions across a concentration range of 5–1000 μg/mL, each exhibiting excellent linearity (R^2^ > 0.999). To assay the nanoparticles, a 100 μL aliquot was thoroughly disrupted in 2.9 mL of methanol by sonication. The drug concentration in the resulting solution was determined by HPLC by interpolating the peak area against the corresponding standard curve. The drug loading efficiency (LE) and encapsulation efficiency (EE) were subsequently calculated using Equations (1), (2).

(1) $$\text{LE}\left(\%\right)=\frac{\text{mass}\;\text{of}\;\text{drug}\;\text{encapsulated}\;\text{in}\;\text{liposomes}}{\text{mass}\;\text{of}\;\text{all}\;\text{materials}\;\text{of}\;\text{liposomes}}\times 100\%$$

(2) $$\text{EE}\left(\%\right)=\frac{\text{mass}\;\text{of}\;\text{drug}\;\text{encapsulated}\;\text{in}\;\text{liposomes}}{\text{mass}\;\text{of}\;\text{drug}\;\text{in}\;\text{liposome}\;\text{suspension}}\times 100\%$$

### 2.6 Collagenase activity

A rhodamine B-labeled collagen substrate (Col-RhB) was first synthesized via EDC/NHS chemistry. Briefly, rhodamine B (24 mg) was activated with NHS (230 mg) and EDC (155 mg) in DMSO (5 mL) for 30 min. This solution was then added to collagen (100 mg) dissolved in 0.1 M MES buffer, and the reaction proceeded for 24 h at room temperature. The conjugate was purified by dialysis (MWCO: 3.5 kDa) against water and lyophilized. Enzyme activity was assessed by incubating Col-RhB (1 mg/mL) with native ColE (1 mg/mL), DSPE-PEG-ColE conjugate (1 mg/mL), or CMPNPs (1 mg/mL) at 37°C with shaking. Aliquots were taken at 0.5, 2, 6, and 12 h, centrifuged, and the absorbance of the supernatant at 560 nm was measured to quantify the release of rhodamine B-labeled collagen, indicative of collagen degradation.

### 2.7 ColE conjugation efficiency

The collagenase conjugation efficiency of CMPNPs was determined using a BCA protein assay kit. Unconjugated collagenase was separated from CMPNPs using 100 kDa ultrafiltration centrifugal tubes by centrifugation at 3500 × g for 30 min, and the filtrate was collected. A standard curve was established with BSA. Briefly, 20 μL of the filtrate was mixed with 200 μL of BCA working reagent and incubated at 37°C for 30 min, and the absorbance was measured at 562 nm to quantify the free (unconjugated) collagenase in the filtrate. ColE Conjugation Efficiency (%) was calculated using Equation (3).

(3) $$\text{Conjugation}\;\text{efficiency}\;\left(\%\right)=\frac{m_{1}-m_{2}}{m_{c}}\times 100\%$$

where m_1_, m_2_, and m_0_ denote the input ColE mass, free ColE mass, and CMPNPs liposome mass, respectively.

### 2.8 Cell culture and viability

A549, MLg, and Raw 264.7 cell lines were cultured at 37°C under 5% CO_2_. A549 and MLg cells were maintained in DMEM/F-12 medium, while Raw 264.7 cells were cultured in MEM. All media were supplemented with 10% fetal bovine serum and 1% penicillin-streptomycin. Cells were routinely passaged upon reaching 80–90% confluency using trypsin-EDTA.

The cytotoxicity of nanoparticles was evaluated in MLg cells using a CCK-8 assay. MLg cells were seeded in 96-well plates (1 × 10^4^ cells/well) overnight. Cells were then treated with various liposomal formulations (BNPs, MNPs, PNPs, MPNPs, CMPNPs) at concentrations ranging from 50 to 600 μg/mL for 24 h. After treatment, cell viability was assessed using a Cell Counting Kit-8 (CCK-8) according to the manufacturer's protocol. Briefly, the medium was replaced with a 10% (v/v) CCK-8 reagent mixture, incubated for 2 h at 37°C, and the absorbance at 450 nm was measured. Relative cell viability (%) was calculated using Equation (4).

(4) $$\text{Viability}\;\left(\%\right)=\frac{{\text{OD}}_{x}-{\text{OD}}_{\text{blank}}}{{\text{OD}}_{100\%}-{\text{OD}}_{\text{blank}}}\times 100\%$$

where OD_x_, OD_blank_, and OD_100%_ represent the absorbance of the treatment group, blank control (medium without cells), and untreated control group (cells without nanoparticles), respectively.

### 2.9 Collagen barrier penetration

The ability of nanoparticles to penetrate a collagenous barrier was assessed using a Transwell system. MLg cells were seeded in the lower chamber (8 × 10^4^ cells/well). Concurrently, a collagen barrier was formed by adding 50 μL of collagen solution (1 mg/mL) to the upper chamber and allowing it to gel. After 12 h, the medium in the upper chamber was replaced with fresh medium containing fluorescent nanoparticles (Cy5.5-labeled BNPs, 100 μg/mL). Following a 6-h co-culture, cells were processed for imaging or quantification. For confocal microscopy, cells were fixed, stained with Hoechst 33342, and imaged. For parallel quantification by flow cytometry, the assay was performed identically using C6-labeled BNPs, after which cells were harvested to analyze cellular fluorescence intensity.

### 2.10 Nanoparticle internalization and distribution

To quantify nanoparticle internalization, MLg cells were plated in 12-well plates at a density of 1 × 10^5^ cells per well. After 12 h of adherence, cells were exposed to C6-fluorescently labeled CMPNPs across a concentration gradient (50, 100, 200, 400, 600 μg/mL) for 6 h. Cells were then washed with cold PBS, detached using trypsin-EDTA, and collected by centrifugation (300 × g, 5 min). The cell pellet was resuspended in PBS containing 1% BSA and analyzed immediately by flow cytometry. Mean fluorescence intensity (MFI) was used as a quantitative measure of cellular association/uptake.

For spatial localization studies, MLg cells were seeded as described and treated with 100 μg/mL of C6-CMPNPs for 6 h. Following treatment, cells were rigorously washed with PBS to remove extracellular nanoparticles, fixed with 4% paraformaldehyde for 15 min at room temperature, and permeabilized with 0.1% Triton X-100. Nuclei were counterstained with Hoechst 33342. Confocal imaging was performed using appropriate laser lines for C6 (E_x_/E_m_ 450/505 nm) and Hoechst (E_x_/E_m_ 346/461 nm).

### 2.11 Wound healing (scratch) assay

We employed a scratch wound assay to evaluate the inhibitory effect of liposomal formulations on TGF-β–induced MLg cell migration. Cells were seeded in 12-well plates. After 12 h, they were pretreated with TGF-β (10 ng/mL) to induce a pro-migratory phenotype, followed by incubation with the respective nanodrugs for 4 h. A uniform scratch was then created in each monolayer using a sterile pipette tip. After washing, cells were maintained in serum-free medium containing TGF-β (10 ng/mL) for 24 h. Wound closure was monitored by imaging at 0 and 24 h. The relative wound area was quantified using Equation (5).

(5) $$\text{Migration}\;\text{rate}\;\left(\%\right)=\frac{W_{0}-W24}{{\Delta W}_{\text{NC}}}\times 100\%$$

where W_0_ and W_24_ are the wound areas at 0 and 24 h, respectively, and ΔW_NC_ is the wound closure of the negative control (TGF-β only).

### 2.12 Migration assay

The effect of liposomal formulations on MLg cell migration was evaluated using a Transwell assay. Briefly, MLg cells (1 × 10^4^ cells/well) were seeded in the upper chamber of Transwell inserts in serum-free medium containing the respective nanomedicines (MNPs, PNPs, MPNPs, CMPNPs). After 6 h, the lower chamber was filled with complete medium containing TGF-β1 (10 ng/mL) as a chemoattractant. Following a 24-h migration period, cells on the upper surface of the membrane were gently removed. The migrated cells on the lower surface were fixed with 4% formaldehyde, stained with 0.1% crystal violet, and imaged under an optical microscope for quantification.

### 2.13 RNA extraction and real-time quantitative PCR (qPCR)

Total RNA was extracted from A549 cells, MLg cells (1 × 10^5^), or lung tissue (5 mg) using a commercial kit (FastPure Cell/Tissue Total RNA Isolation Kit V2, Vazyme). RNA concentration was measured with a NanoDrop One spectrophotometer. For cDNA synthesis, 1 μg of total RNA was reverse-transcribed using the HiScript II 1st Strand cDNA Synthesis Kit (Vazyme) with a gDNA wiper step. Quantitative PCR was performed on a QuantStudio™ 5 Real-Time PCR System using ChamQ SYBR qPCR Master Mix (Vazyme) in a 20 μL reaction volume. The thermal cycling protocol was: 95°C for 30 s, followed by 40 cycles of 95°C for 10 s and 60°C for 30 s, with a final melt curve stage. Gene expression was calculated using the 2^−ΔΔCt^ method and normalized to a housekeeping gene. Primer sequences are listed in Table S1.

### 2.14 Western blot

Proteins were extracted from cell pellets (2 × 10^5^ cells) or homogenized tissues (20 mg) using RIPA lysis buffer supplemented with 1 mM PMSF and protease inhibitors. Protein concentration was determined by a BCA assay and equal amounts of protein were denatured at 95°C for 10 min. Samples were separated by SDS-PAGE on 10% or 12% gels and electrophoretically transferred onto 0.45 μm PVDF membranes. Membranes were blocked with 5% skim milk in TBST for 2 h at room temperature, followed by incubation with primary antibodies overnight at 4°C and corresponding HRP-conjugated secondary antibodies for 2 h at room temperature. Protein bands were visualized using enhanced chemiluminescence (ECL) substrate and imaged with an Amersham ImageQuant 800 system (Cytiva).

### 2.15 IPF mouse model and in vivo treatment

All animal procedures were approved by the Animal Care and Use Committee of Wannan Medical College (Approval No. WNMC-AWE-2024358) and complied with relevant ethical guidelines. An IPF model was established in male C57BL/6 mice (18–22 g) by a single intratracheal instillation of bleomycin (BLM, 2 mg/kg in 75 μL sterile saline) under sodium amobarbital anesthesia. For the therapeutic study, mice received 100 μL of the respective liposomal formulations via inhalation on days 3, 5, 7, 9, 11, 13, and 15 post-BLM injury, delivering equivalent doses of 2 mg/kg PFD and 1.8 mg/kg MPS. Body weight was monitored throughout the study. On day 21, mice underwent in vivo lung CT imaging before sacrifice. Lung tissues were harvested for molecular, cellular (flow cytometry), and histopathological analyses. Blood was collected via cardiac puncture for systemic cytokine profiling.

### 2.16 In vivo and ex vivo fluorescence imaging

The biodistribution of nanoparticles administered via different routes was tracked using fluorescence imaging. Mice received 50 μL of Cy5.5-labeled CMPNPs either by intravenous (i.v.) injection or by inhalation administration. Whole-body fluorescence images of anesthetized mice were acquired at 15 min, 1-, 6-, and 24-h post-administration using an IVIS Lumina LT system (PerkinElmer). Subsequently, mice were euthanized, and major organs (lungs, liver, spleen, kidneys, heart) were harvested for ex vivo imaging to quantify tissue-specific accumulation.

### 2.17 CT imaging

Pulmonary structure and fibrosis were assessed non-invasively by micro-computed tomography (μCT). Mice were scanned under anesthesia using a Hiscan XM system (Suzhou Hiscan Information Technology) with the following parameters: X-ray tube voltage 60 kV, current 133 μA, exposure time 1.2 s per projection, and a voxel size of 50 μm. A full 360° rotation was performed with a step size of 0.5°. The acquired projection images were reconstructed into three-dimensional volumes using the vendor's Hiscan Reconstruct software (v3.0). Subsequent quantitative analysis of lung density and volume was conducted using the Hiscan Analyzer software (v3.0).

### 2.18 Histological staining

For histopathological evaluation, harvested lung tissues were immediately fixed in 4% paraformaldehyde for 48 h at 4°C. Following standard dehydration and clearing steps, tissues were embedded in paraffin blocks. Serial sections (4–5 μm thickness) were prepared. H&E staining, Masson's trichrome staining, and immunohistochemical (IHC) staining were performed according to standard protocols by a professional service provider (Wuhan Servicebio Technology Co., Ltd.). Pulmonary fibrosis was semi-quantified by scoring Masson's trichrome-stained sections according to the modified Ashcroft scale [35].

### 2.19 Flow cytometric analysis

To obtain high-viability single-cell suspensions, freshly harvested lungs were processed immediately. Tissues were minced and digested in a solution of Collagenase I (1 mg/mL) and DNase I (0.1 mg/mL) in PBS at 37°C for 40 min with periodic trituration. The reaction was halted with an equal volume of ice-cold RPMI-1640 medium supplemented with 5% fetal bovine serum (FBS). The cell suspension was filtered (70 μm), centrifuged (300 × g, 5 min, 4°C), and washed twice to remove debris.

Immunophenotyping was performed using a standardized 6-color surface marker panel. Briefly, 1 × 10^6^ cells per sample were first stained for viability using a fixable LIVE/DEAD™ Near-IR viability dye (Thermo Fisher) for 30 min at room temperature. Non-specific binding was blocked with purified anti-mouse CD16/32 antibody (TruStain FcX™, BioLegend) for 10 min. For surface marker staining, cells were incubated with a cocktail of fluorescently conjugated antibodies (anti-mouse CD14-Human, Catalog: E-AB-F1209C, anti-mouse CD45-PE, Catalog: E-AB-F1136D, anti-mouse Ly6G-PE, Catalog: E-AB-F1108D, and anti-mouse/HumanCD11b-FITC, Catalog: E-AB-F1081C, anti-mouse F4/80-PE, Catalog: E-AB-F0995D, anti-mouse CD206-APC, Catalog: E-ABF1135E, anti-mouse CD86-FITC, Catalog: E-AB-F0994C) at 4°C in the dark for 15 min. After final washes, cells were resuspended in 500 μL of staining buffer. Data were acquired on a FACSVerse™ systems (BD Biosciences). Compensation was performed using single-stained compensation beads, and fluorescence minus one (FMO) control were used to guide accurate gating. Data analysis was conducted using FlowJo software.

### 2.20 Hydroxyproline measurement

Lung collagen deposition was quantified by measuring hydroxyproline (HYP) levels using a commercial assay kit (Nanjing Jiancheng Bioengineering Institute). Approximately 20 mg of lung tissue was hydrolyzed, and the HYP content was determined according to the manufacturer's instructions. Briefly, the hydrolysate was oxidized and reacted with a chromogenic substrate, and the absorbance was measured at 550 nm. The HYP concentration was calculated using a standard curve and expressed as micrograms per milligram of wet lung tissue.

### 2.21 Plasma biochemical analysis

Systemic biochemical parameters, including aspartate aminotransferase (AST), alanine aminotransferase (ALT), creatinine (CRE), and blood urea nitrogen (BUN), were measured in plasma using commercial colorimetric assay kits (Nanjing Jiancheng Bioengineering Institute). All assays were performed in strict accordance with the manufacturer's protocols. As a representative example, for the AST assay, plasma samples were incubated with the specific substrate at 37°C for 30 min. The reaction was then developed by sequential addition of 2,4-dinitrophenylhydrazine and sodium hydroxide. After a 15-min incubation, the absorbance at 510 nm was measured, and the enzyme activity (U/L) was calculated by interpolation from a standard curve run in parallel.

### 2.22 Statistical analysis

All statistical data were expressed as mean ± SD at least in triplicate each time. Differences between the two groups were analyzed using ANOVA or Student's t-test. The difference was considered statistically significant at *p ≤ 0.05, **p ≤ 0.01, and ***p ≤ 0.001.

## 3 Results and discussion

### 3.1 Preparation and characterization of nanomedicines

Collagenase-modified DSPE-PEG (DSPE-PEG-ColE) was first synthesized via an EDC-mediated condensation reaction. Successful conjugation and retained enzymatic activity were confirmed by infrared spectroscopy, UV-vis absorption, and collagen degradation assays (Fig. S1–S3). Subsequently, liposomal nanomedicines were prepared using a classical formulation consisting of cholesterol, DOPE, and either unmodified DSPE-PEG or DSPE-PEG-ColE (for collagenase-coated formulations) at a molar ratio of 38.5:10:1.5. To ensure stability and uniform size distribution, the synthesis was carried out via ethanol injection using a microfluidic system, yielded a series of nanoparticles: blank liposomes (BNPs), MPS-loaded nanoparticles (MNPs), PFD-loaded nanoparticles (PNPs), dual-loaded nanoparticles (MPNPs), and ColE-modified MPNPs (CMPNPs). Hydrodynamic particle size measurements showed that the diameters of BNPs, MNPs, PNPs, MPNPs, and CMPNPs were 100.76, 156.61, 153.32, 161.43, and 232.80 nm, respectively (Fig. 1a). The polydispersity index ranged from 0.128 to 0.193, indicating a relatively narrow size distribution. The zeta potentials of all nanoparticle formulations ranged from −5.84 to −9.23 mV (Fig. 1b). This slightly negative surface charge is expected to minimize adhesion to the mucus layer lining the lung epithelium and enhance mucus penetration by reducing electrostatic interactions with negatively charged mucin glycoproteins, thereby favoring pulmonary delivery via inhalation administration [36].

**Fig. 1:**
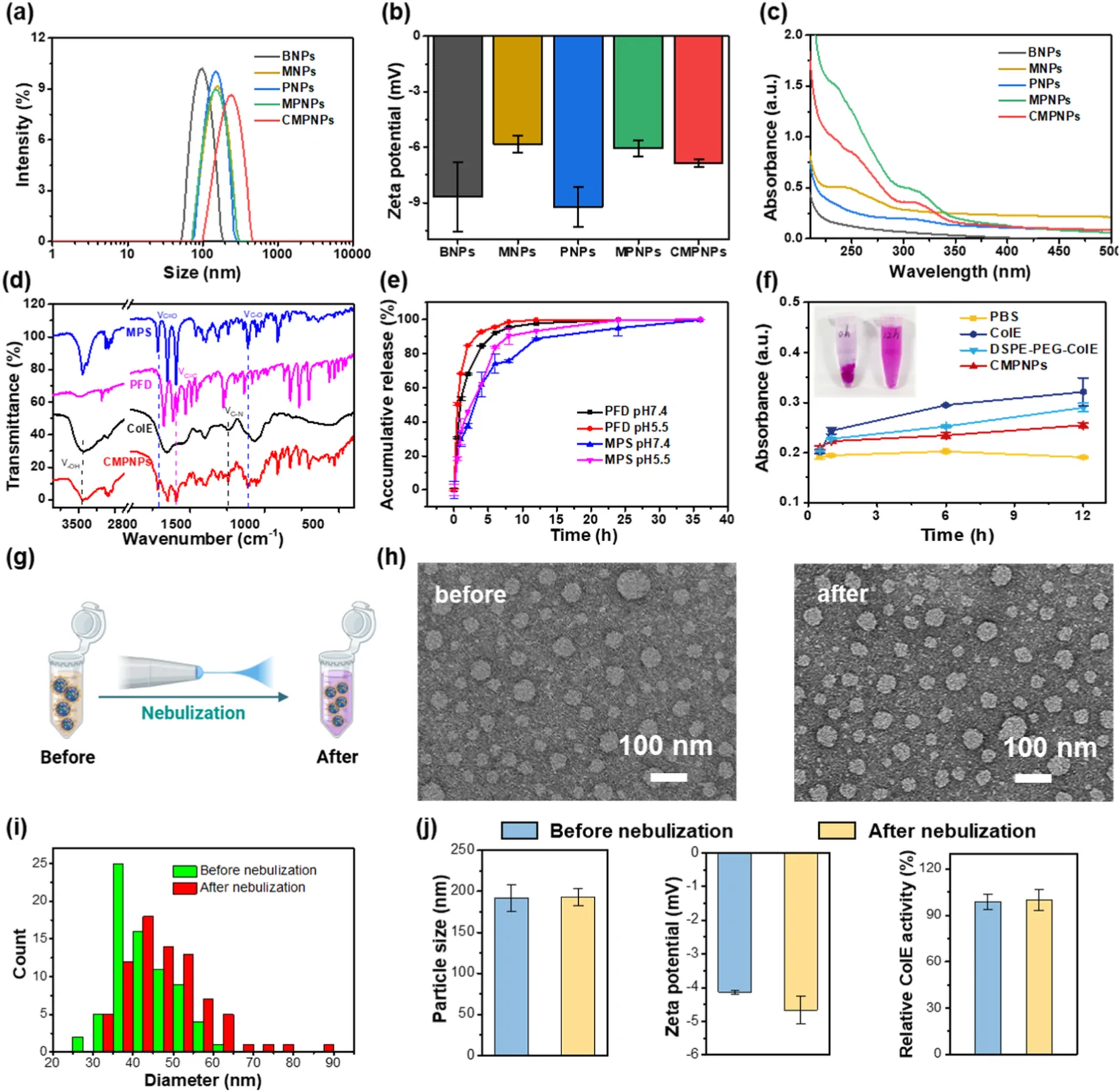
Comprehensive characterization of the developed nanomedicines. The physicochemical properties of all nanoparticle formulations (BNPs, MNPs, PNPs, MPNPs, and CMPNPs) were systematically assessed, including (a) hydrodynamic size distribution, (b) zeta potential, and (c) UV-vis absorption spectra. (d) Chemical composition was verified by FTIR spectroscopy for individual components (MPS, PFD, ColE) and the final CMPNPs. (e) The in vitro release kinetics of MPS and PFD from CMPNPs under acidic (pH 5.5) and physiological (pH 7.4) conditions, and (f) the collagen degradation capability of free ColE versus CMPNPs. (g) Schematic of the sampling scheme for CMPNPs before and after nebulization. (h) TEM images of CMPNPs and (i) corresponding size distribution before and after nebulization. (j) Hydrodynamic diameter, zeta potential, and collagenase activity of CMPNPs before and after nebulization. Data are shown as mean ± SD (n ≥ 3).

UV–vis absorption spectroscopy was performed to verify the successful incorporation of individual components into the nanoparticles. As shown in Fig. 1c and Fig. S4, CMPNPs exhibited characteristic absorption peaks at positions 243, 259, and 320 nm, corresponding to MPS, ColE, and PFD, respectively. Moreover, distinct peaks originating from ColE, MPS, and PFD were observed in the Fourier-transform infrared (FTIR) spectra of CMPNPs: ν_–OH_ at 3421 cm^−1^, ν_C=O_ at 1711 cm^−1^, ν_C=C_ at 1594 cm^−1^, ν_C–N_ at 1241 cm^−1^, and ν_C–O_ at 1110 cm^−1^ (Fig. 1d). These results collectively confirm the successful incorporation of the intended functional components into each nanoparticle formulation. In addition, the colloidal stability of the nanoparticles was further evaluated by monitoring their hydrodynamic diameter in PBS, complete culture medium (10% FBS) and DI water using dynamic light scattering (DLS). No significant aggregation was observed in any of the three media, indicating good colloidal stability (Fig. S5).

The drug loading efficiency (LE) and encapsulation efficiency (EE) of MPS and PFD in CMPNPs were determined by high-performance liquid chromatography (HPLC) following DMSO-mediated lysis. For MPS, the LE and EE were 5.46% and 29.50%, respectively; for PFD, the LE and EE were 3.19% and 17.26%, respectively. The collagenase conjugation efficiency of CMPNPs was determined to be 16.12% by the BCA assay [37]. The release kinetics of MPS and PFD from CMPNPs were examined at pH 5.5 and pH 7.4. As shown in Fig. 1e, both drugs were released rapidly, with cumulative release exceeding 80% within 10 h. Release proceeded slightly faster at pH 5.5, which may arise from protonation-induced destabilization of the DOPE membrane under acidic conditions [38]. Furthermore, the collagenase activity of surface-modified ColE was confirmed using a collagen degradation assay (Fig. 1f) [39]. Free ColE, DSPE-PEG-ColE, and CMPNPs all degraded collagen in a time-dependent manner. These results verify that the conjugated enzyme retains potent biological activity, supporting its potential utility to overcome the pathological collagen barrier.

Given that shear forces during atomization may compromise nanoparticle integrity [40], we compared the physicochemical and functional properties of CMPNPs before and after nebulization (Fig. 1g). Transmission electron microscopy (TEM) analysis revealed that the nanoparticles retained intact phospholipid bilayers following nebulization, with no observable changes in particle morphology (Fig. 1h). A modest widening of the size distribution was noted post-nebulization (Fig. 1i), likely attributable to minor particle rearrangement induced by air–liquid interface forces during atomization. As shown in Fig. 1j and Fig. S6a, nebulization did not significantly affect the size, zeta potential, collagenase activity, or UV–vis spectra of CMPNPs, indicating preserved colloidal stability and bioactivity under shear stress. The other formulations (BNPs, MNPs, PNPs, and MPNPs) similarly retained stable size, PDI, and zeta potential after nebulization (Fig. S6b–d), further confirming their robustness against nebulization-induced shear forces.

### 3.2 Cell uptake and penetration of the collagen barrier

Before proceeding to intracellular transport studies, the cytotoxicity of the nanomedicines was evaluated against three representative cell lines: A549 (modeling alveolar epithelial type II cells), MLg (lung fibroblasts), and Raw264.7 (macrophages). Using the CCK-8 assay, cell viability remained above 85% for all cell types at concentrations up to the highest tested concentration of 600 μg/mL, indicating negligible cytotoxicity (Fig. 2a). To assess cellular uptake and distribution, MLg cells were incubated with Coumarin 6 (C6)-labeled CMPNPs. Confocal laser scanning microscopy (CLSM) revealed a uniform cytoplasmic distribution of the fluorescence signal, indicating effective intracellular delivery (Fig. S7). To quantify this uptake, cells were incubated with varying concentrations of C6-CMPNPs for 6 h, and flow cytometric analysis demonstrated a concentration-dependent increase in cellular fluorescence intensity (Fig. 2b and c). Accordingly, 200 μg/mL was chosen as the optimal dose for subsequent in vitro studies based on the dose–response relationship and cytotoxicity data.

**Fig. 2:**
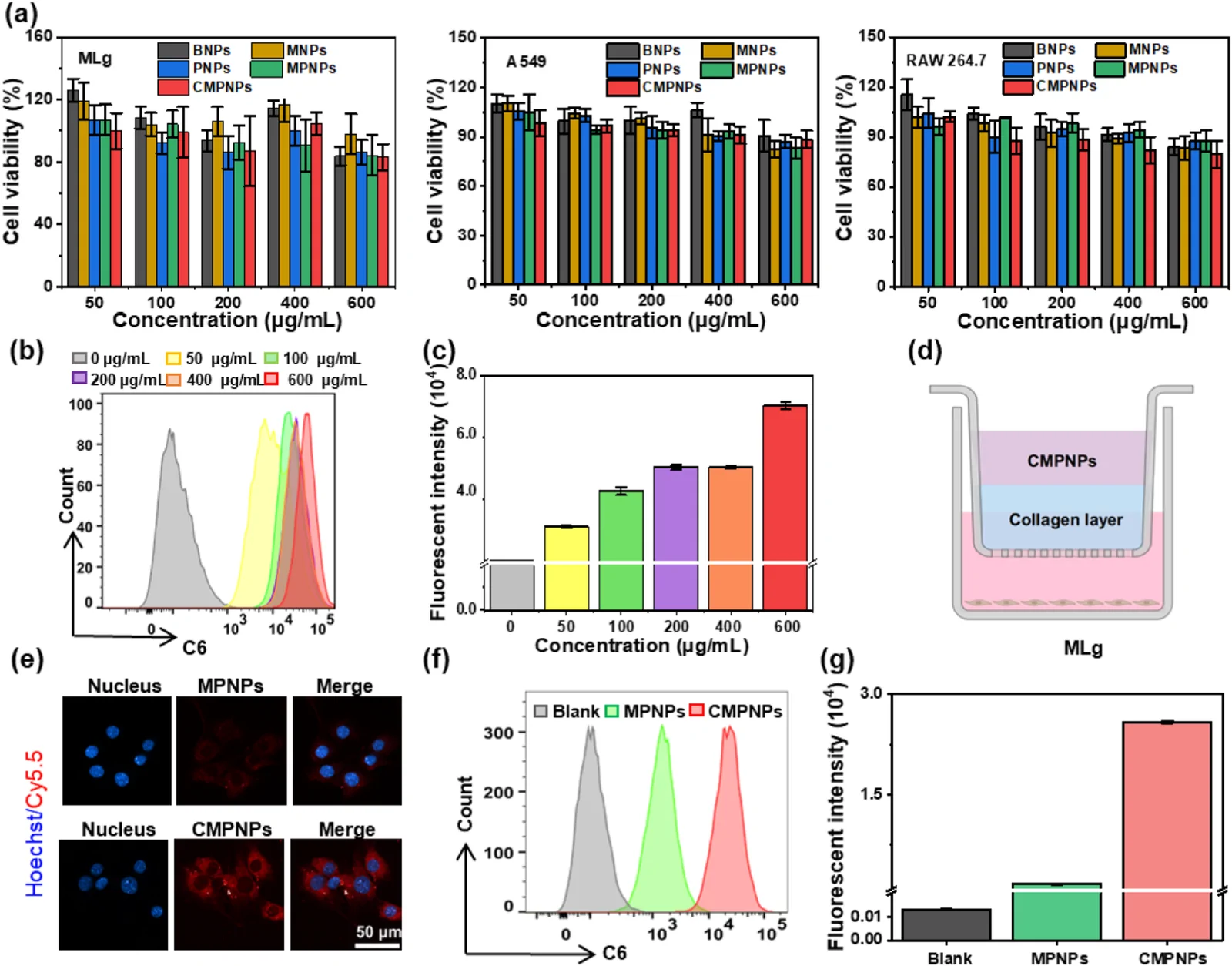
(a) Cytotoxicity profiles of CMPNPs in MLg, A549, and RAW 264.7 cells. (b) Concentration-dependent cellular uptake of CMPNPs quantified by flow cytometry and (c) corresponding quantitative statistics. (d) Schematic illustration of the Transwell-based collagen barrier model. (e) Laser confocal microscopy images depicting the uptake of MPNPs or CMPNPs by MLg cells following penetration through the collagen barrier. (f) Flow cytometric analysis of MLg cell uptake of MPNPs or CMPNPs under the collagen barrier, and (g) corresponding quantitative statistical results. Data are shown as mean ± SD (n ≥ 3).

To evaluate the barrier-penetrating capability conferred by ColE modification, an in vitro collagen barrier model was established using a Transwell system (Fig. 2d), with CMPNPs placed in the upper collagen-coated chamber and MLg cells seeded in the lower chamber as the target. Confocal microscopy revealed a markedly stronger intracellular fluorescence signal for CMPNPs than for non-enzymatic MPNPs (Fig. 2e). Flow cytometric quantification corroborated this result, showing an approximately fourteen-fold increase in cellular fluorescence for the ColE-modified nanoparticles over the unmodified control (Fig. 2f and g). These data indicate that surface-anchored ColE degrades the collagen barrier enzymatically and thus promotes nanoparticle penetration through dense collagen matrices [41]. It should be noted that this collagen-coated Transwell model is a simplified mimic of the fibrotic barrier. The lung microenvironment in IPF is far more complex, containing a viscous mucus layer and other ECM components in addition to dense collagen [42]. The penetration enhancement observed here should therefore be viewed as a proof of concept for collagenase-mediated barrier traversal, and its magnitude in the diseased lung may differ from that measured in this model.

### 3.3 Inhibition of TGF-β1–induced myofibroblast differentiation

Myofibroblast activation and aberrant ECM deposition are pivotal drivers of lung structural remodeling in IPF. To investigate how CMPNPs counteract this process, we established a TGF-β1-stimulated in vitro fibrosis model using MLg cells (Fig. 3a) [43]. Following stimulation with TGF-β1, cells were treated with CMPNP. At the transcriptional level, CMPNPs significantly blunted the TGF-β1-induced expression of fibrosis markers associated with myofibroblast differentiation (ACTA2) and matrix deposition (FN and Col1a1) (Fig. 3b). This suppression was corroborated at the translational level, as Western blot analysis showed that CMPNPs pretreatment effectively inhibited the TGF-β1-induced upregulation of α-SMA and COL1A1 proteins (Fig. 3c and d). These findings mechanistically demonstrate that CMPNPs suppress myofibroblast transdifferentiation and ECM overproduction, accounting for their anti-fibrotic activity.

**Fig. 3:**
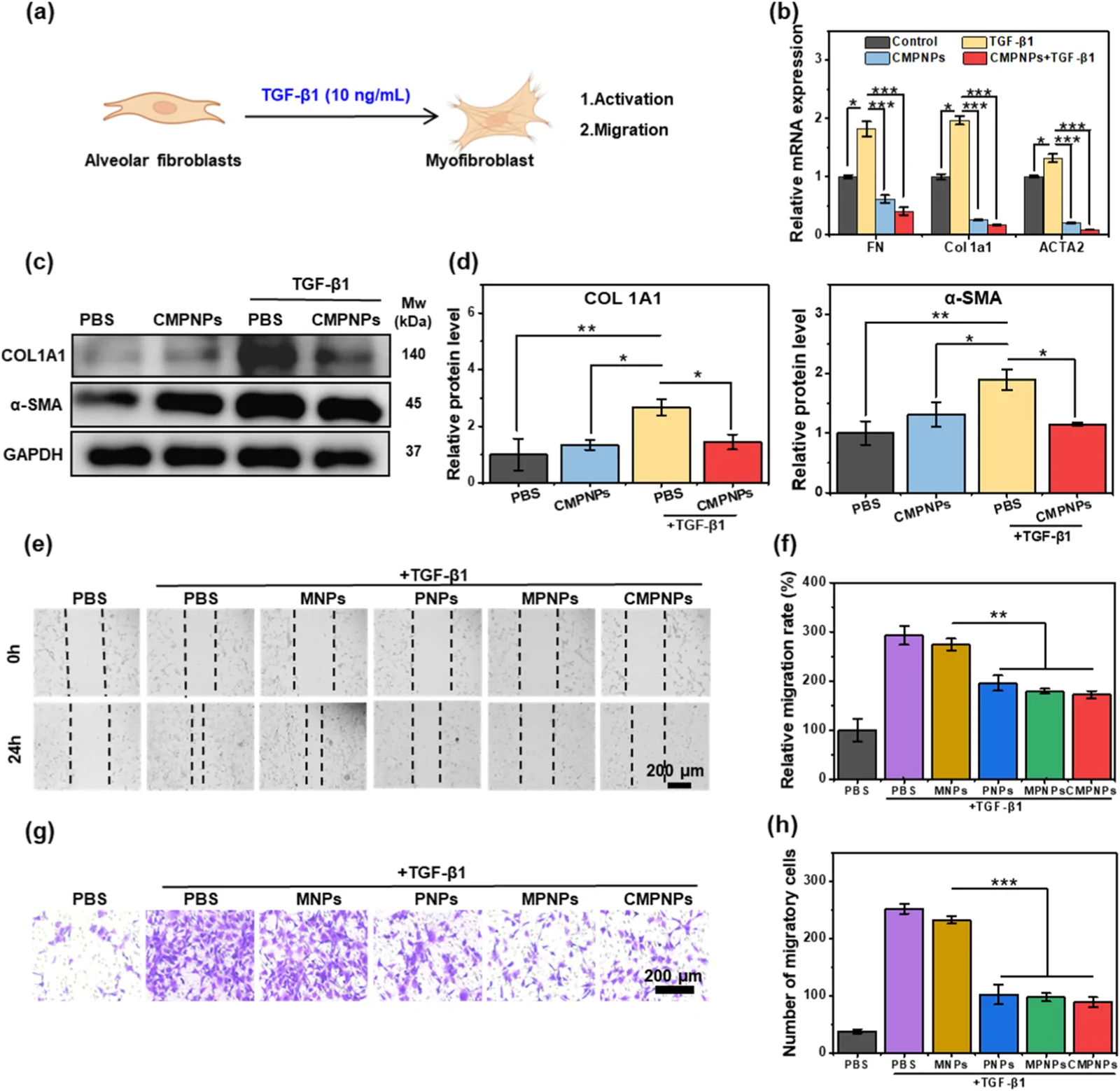
CMPNPs inhibit TGF-β1–induced myofibroblast differentiation. (a) Schematic illustration of the experimental paradigm. (b) qPCR analysis of mRNA expression levels of fibrosis-associated markers across experimental groups. (c) Representative Western blot images and (d) corresponding densitometric quantification of COL1A1 and α-SMA protein levels. (e) Phase-contrast images of scratch wounds immediately after creation (0 h) and after 24 h of TGF-β1 stimulation, and (f) the corresponding quantification of relative wound area. (g) Representative micrographs of crystal violet-stained migrated cells in a Transwell assay, and (h) the corresponding quantification of cell counts. Data are presented as mean ± SD (n ≥ 3). Statistical significance is indicated as *P < 0.05, **P < 0.01, and ***P < 0.001.

The recruitment and migration of fibroblasts to sites of alveolar injury represent a critical early step in pulmonary fibrogenesis [44]. Therefore, we evaluated the ability of the nanoparticle formulations to interfere with this process using wound healing and Transwell migration assays. Consistent with previous reports, TGF-β1 stimulation significantly enhanced the migration of MLg cells. Notably, all nanoparticle formulations encapsulating the anti-fibrotic drug pirfenidone (PFD), i.e., PNPs, MPNPs, and CMPNPs, exerted the strongest inhibitory effect on TGF-β1-driven cell migration (Fig. 3e–h). In contrast, BNPs and MNPs showed minimal or no inhibition, confirming that PFD is the principal mediator of the anti-migratory activity within the combinatorial nanomedicines [45], [46]. Collectively, these results demonstrate that CMPNPs target fibroblast recruitment and thus hold therapeutic promise for restraining fibrotic progression in IPF.

### 3.4 Modulation of macrophage polarization in vitro

During the onset and progression of IPF, M1 macrophages become aberrantly activated in the early disease stage. These cells secrete pro-inflammatory factors, including interleukin-1β (IL-1β) and tumor necrosis factor-α (TNF-α), as well as reactive oxygen and nitrogen species, thereby contributing to persistent alveolar epithelial injury [47]. This process initiates and amplifies local inflammation, creating a microenvironment conducive to subsequent fibrogenesis [48]. To elucidate the regulatory effects of CMPNPs on M1 polarization, macrophages were first induced toward the pro-inflammatory M1 phenotype using lipopolysaccharide (LPS, 100 ng/mL) combined with interferon-γ (IFN-γ, 20 ng/mL) (Fig. 4a). As shown in Fig. 4b, under these inducing conditions, the mRNA levels of M1 markers, including inducible nitric oxide synthase (iNOS), cluster of differentiation 86 (CD86), and the pro-inflammatory cytokine IL-1β, increased by 38.0, 25.9, and 23.7-fold, respectively, confirming successful M1 polarization induction. Notably, subsequent treatment with CMPNPs significantly reduced these markers. Western blot analysis (Fig. 4c) and its corresponding densitometric quantification (Fig. 4d) further corroborated these findings. Flow cytometry was additionally employed to quantify the proportion of M1 macrophages. As depicted in Fig. 4e and f, LPS/IFN-γ treatment increased the M1 macrophage ratio to 23.5%, whereas CMPNP treatment reduced it to 11.3%. Collectively, these results demonstrate that CMPNPs selectively inhibit M1 polarization, suggesting their potential to modulate macrophage immune status, influence inflammatory cell recruitment, and attenuate inflammatory responses.

**Fig. 4:**
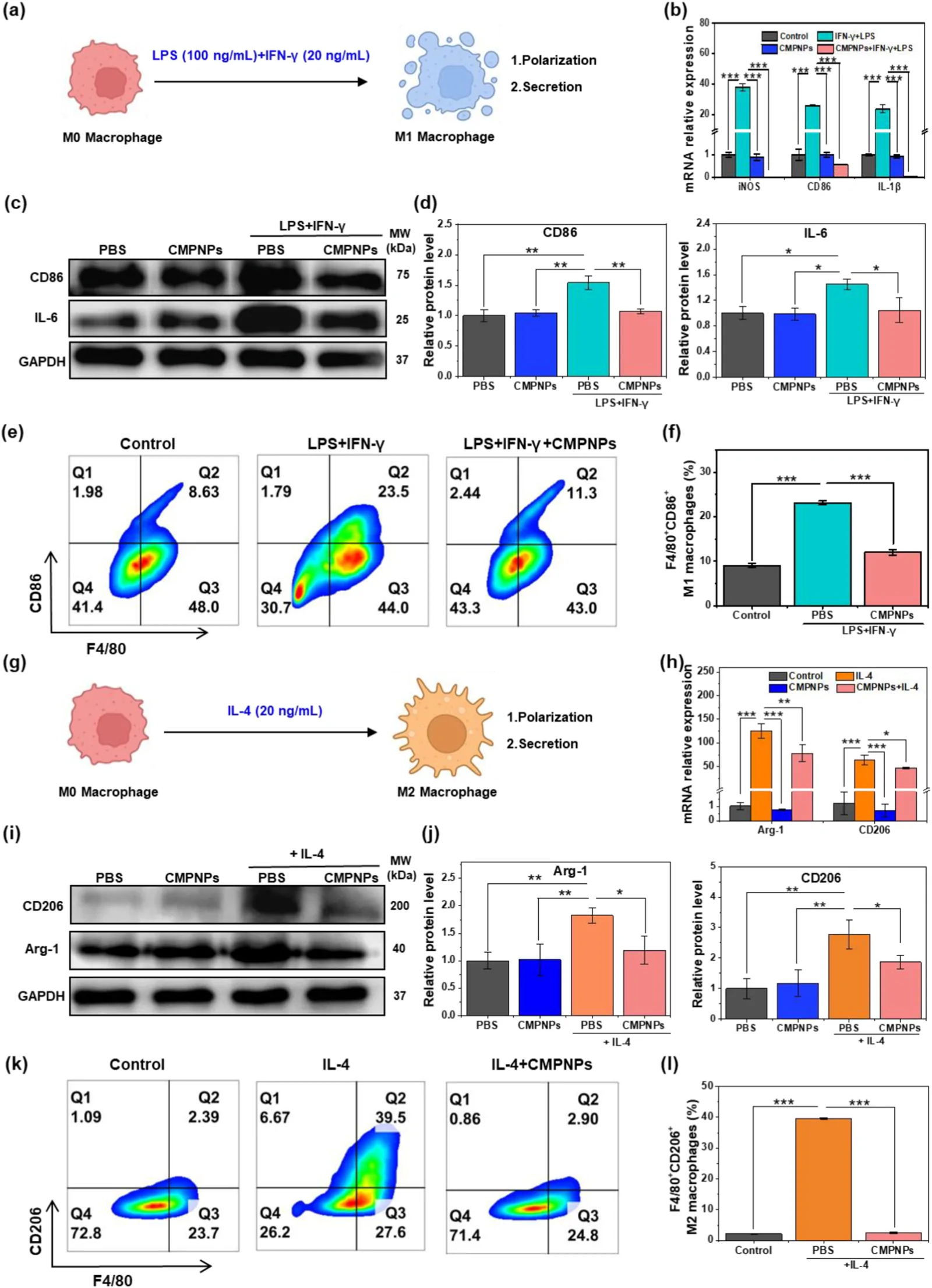
CMPNPs regulate M1/M2 macrophage polarization in vitro. (a) Schematic illustration of M0 macrophage polarization toward the pro-inflammatory M1 phenotype upon LPS stimulation. (b) Relative mRNA expression levels of M1-associated markers (iNOS, CD86, IL-1β). (c) Representative Western blots and (d) quantitative analysis of CD86 and IL-6 protein levels. (e) Flow cytometric analysis of M1 macrophages (F4/80^+^CD86^+^). (f) Quantification of the proportion of F4/80^+^CD86^+^ M1 cells. (g) Schematic illustration of M0 macrophage polarization toward the pro-fibrotic M2 phenotype upon IL-4 stimulation. (h) Relative mRNA expression levels of M2-associated markers (Arg-1, CD206). (i) Representative Western blots and (j) quantitative analysis of CD206 and Arg-1 protein levels. (k) Flow cytometric analysis of M2 macrophages (F4/80^+^CD206^+^). (l) Quantification of the proportion of F4/80^+^CD206^+^ M2 cells. Data are expressed as mean ± SD (n ≥ 3); *P < 0.05, **P < 0.01, ***P < 0.001.

During the progressive fibrotic phase, excessive M2 macrophage polarization becomes predominant. These cells play a critical role in fibrogenesis by secreting pro-fibrotic mediators such as TGF-β and platelet-derived growth factor (PDGF), which aberrantly activate fibroblasts, promoting their proliferation and transdifferentiation into collagen-producing myofibroblasts [49], [50], [51]. Therefore, the ability of CMPNPs to modulate M2 polarization was evaluated using an interleukin-4 (IL-4)-induced model (Fig. 4g). As illustrated in Fig. 4h, IL-4 treatment upregulated the mRNA levels of the M2 markers arginase-1 (Arg-1) and CD206. Co-treatment with CMPNPs suppressed the expression of these M2-related genes. This observation was corroborated by Western blot analysis and its densitometric quantification (Fig. 4i and j). IL-4 increased CD206 protein by 2.71-fold and Arg-1 by 1.83-fold, both of which were reversed by CMPNP co-treatment. Flow cytometry (Fig. 4k and l) quantified M2 macrophages (F4/80^+^CD206^+^). IL-4 raised the M2 ratio to 39.5%, whereas CMPNPs reduced it to 2.90%. The anti-fibrotic components of CMPNPs are known to target M2 macrophages: PFD suppresses M2 activation via integrin αMβ2 and ROCK2 [52]. Consequently, CMPNPs are positioned to disrupt the key pathogenic link in which aberrant M2 macrophages drive excessive fibroblast activation, thereby mitigating the dysregulated repair and fibrotic cascade in IPF.

### 3.5 Anti-fibrotic efficacy in the IPF mouse model

A comparative efficacy study was conducted using the bleomycin (BLM)-induced pulmonary fibrosis mouse model [53], with the experimental schema outlined in Fig. 5a. Briefly, three days after BLM instillation, mice were randomized into five treatment groups (n = 6 per group): a vehicle-treated model group (normal saline, NS), four groups receiving intratracheal administration of the respective nanomedicine formulations (MNPs, PNPs, MPNPs, or CMPNPs) every 48 h, and a non-injured healthy control group. On day 21, the NS-treated model group exhibited significant disease progression, as evidenced by sustained body weight loss and a final survival rate of 50% (Fig. 5b and c). Treatment with MNPs, PNPs, or MPNPs conferred a significant survival benefit compared to the model group. Notably, the CMPNPs-treated group achieved complete survival and effectively prevented the weight loss observed in the model group. These findings demonstrate that CMPNPs possess superior efficacy in mitigating BLM-induced mortality and systemic morbidity.

**Fig. 5:**
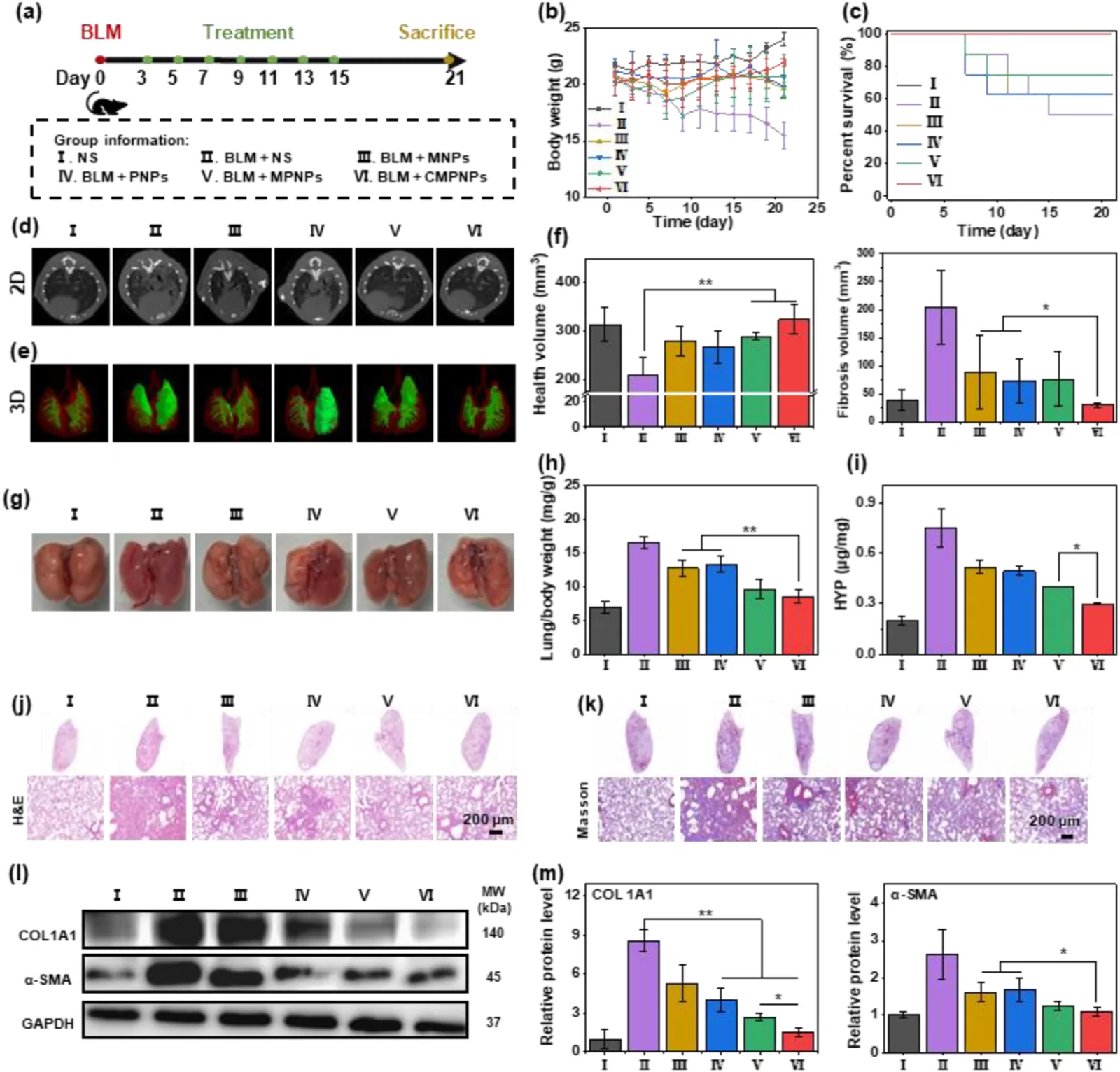
Therapeutic evaluation of liposomal nanodrugs in a BLM-induced IPF mouse model. (a) Schematic illustration of the experimental timeline and group allocation. (b) Body weight curves and (c) survival curves for each treatment group throughout the treatment period in the BLM-induced IPF mouse model. (d) Representative cross-sectional (transverse) CT images of the lungs and (e) 3D reconstructed CT images for each treatment group on day 21 post-BLM induction (burgundy: healthy tissue < 600 HU; green: fibrotic tissue > 600 HU). (f) Quantitative analysis of healthy and fibrotic lung tissue volumes (mm^3^) derived from 3D CT images for each treatment group. (g) Gross photographs of isolated lungs from each treatment group. (h) Lung index (lung weight-to-body weight ratio) across treatment groups. (i) Hydroxyproline (HYP) content in lung tissue across treatment groups. (j, k) Representative images of H&E and Masson's trichrome staining of lung tissue from each treatment group. (l) Representative Western blots of COL1A1 and α-SMA protein levels, and (m) corresponding quantification normalized to GAPDH. Data are presented as mean ± SD (n ≥ 3); *P < 0.05, **P < 0.01, ***P < 0.001.

Pulmonary fibrosis was assessed non-invasively by chest computed tomography (CT) on day 21. BLM-injured mice developed pronounced high-attenuation opacities and reticulation, characteristic of fibrotic remodeling [54]. These abnormalities were most severe in the NS-treated model group (Fig. 5d). For quantification of pulmonary fibrosis, a Hounsfield unit (HU) thresholding algorithm was applied to construct three-dimensional (3D) CT images. Specifically, voxels with attenuation values > 600 HU were segmented and color-coded green to represent fibrotic tissue, whereas voxels with values between 0 and 600 HU were classified as healthy lung parenchyma and burgundy. Anatomical structures with inherently high attenuation (trachea and bronchi) were included in the fibrotic classification to avoid underestimation. As shown in Fig. 5e, compared with the healthy group, the NS group exhibited characteristic architectural remodeling driven by dense fibrosis, frequent honeycombing, and patchy lung involvement, which collectively contributed to the progressive loss of normal lung function. In contrast, treatment with the nanomedicines markedly attenuated fibrosis, with the most pronounced reduction observed in the MPNPs and CMPNPs groups. Subsequent 3D volumetric quantification corroborated these observations (Fig. 5f). The CMPNPs group exhibited the largest volume of healthy lung parenchyma and the smallest fibrotic burden, demonstrating superior therapeutic efficacy.

For comprehensive histopathological assessment on day 21, lung tissues from the IPF model group showed significant injury with hemorrhagic necrosis and inflammatory infiltration, which were substantially improved by nanomedicine treatment (Fig. 5g). The lung-to-body weight index, a measure of inflammation and edema severity, was significantly elevated in the BLM-injured model group (16.55) versus healthy controls (6.82) and was effectively reversed by nanomedicines, with CMPNPs showing the most potent effect (MNPs, 12.78; PNPs, 13.34; MPNPs, 9.60; CMPNPs, 8.54) (Fig. 5h). Histological analysis was performed to assess lung tissue damage and collagen deposition. H&E staining revealed that BLM injury induced severe structural damage, including alveolar collapse and inflammatory cell aggregation, which was substantially alleviated by nanomedicine treatment (Fig. 5j). Masson's trichrome staining further showed a significant reduction in collagen fibers following treatment (Fig. 5k). This reduction was biochemically confirmed by hydroxyproline (HYP) quantification. HYP levels increased sharply in the model group but were suppressed by all nanomedicines, with CMPNPs achieving the greatest suppression (Fig. 5i). These results highlight the enhanced anti-fibrotic efficacy of the combinatorial formulation.

To further assess the anti-fibrotic effects of CMPNPs at the molecular and histological levels, we examined the expression of key fibrotic markers. RT-qPCR showed that BLM injury significantly upregulated the mRNA levels of Col1a1, ACTA2, Fn, and TGF-β1 in lung tissue. Treatment with all drug-loaded liposomes reversed this upregulation, with CMPNPs exerting the most potent inhibitory effect (Fig. S8). Western blot analysis corroborated these findings at the protein level, demonstrating a corresponding decrease in COL1A1 and α-SMA expression (Fig. 5l and m). Immunofluorescence staining further visualized this suppression in situ, showing that the intense interstitial co-localization of α-SMA and COL1A1 in model lungs was markedly diminished, particularly after CMPNPs treatment, which coincided with better-preserved alveolar architecture (Fig. S9). In short, these results demonstrate that CMPNPs effectively disrupts the core fibrotic cascade by concurrently inhibiting myofibroblast differentiation and collagen deposition, offering a potent therapeutic strategy for IPF.

### 3.6 Anti-inflammatory and immunomodulatory effects of nanoparticles in vivo

We further evaluated the inflammatory and immune activation status of BLM-induced model mice following treatment with the nanomedicine formulations. At the transcriptional level, RT-qPCR analysis of lung tissue (Fig. S10) showed that BLM-induced lung injury significantly upregulated the mRNA expression of the pro-inflammatory cytokines interleukin-6 (IL-6), tumor necrosis factor-α (TNF-α), and interleukin-1β (IL-1β), the M1 macrophage marker inducible nitric oxide synthase (iNOS), and the M2 macrophage marker arginase-1 (Arg-1). Notably, this upregulation was attenuated by nanomedicines loaded with MPS or PFD, with the CMPNPs group exhibiting the most pronounced effect. Consistent with the transcriptional findings, immunohistochemical staining of lung sections (Fig. 6a) confirmed that BLM instillation markedly increased the pulmonary protein levels of IL-6, TNF-α, and IL-1β, and that these increases were substantially suppressed by the drug-loaded nanoformulations. Serum ELISA analysis (Fig. 6b) further demonstrated that the nanomedicines significantly reduced circulating levels of IL-6 and TNF-α, indicating effective control of both pulmonary and systemic inflammation.

**Fig. 6:**
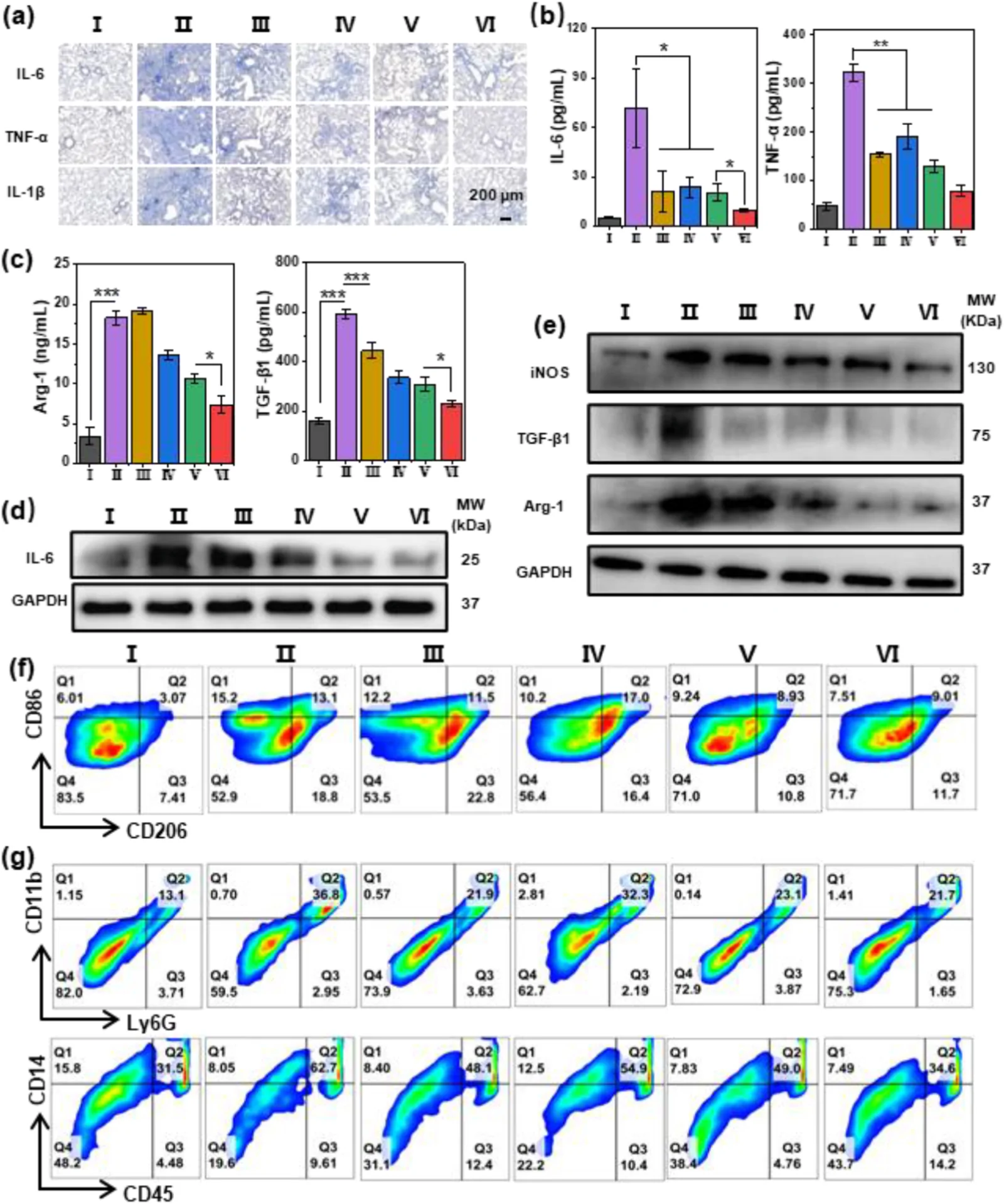
Anti-inflammatory and immunomodulatory effects of liposomal nanodrugs in a BLM-induced IPF mouse model. (a) Immunohistochemical localization of IL-6, TNF-α, and IL-1β in lung tissue sections from different treatment groups. (b, c) Plasma levels of M1-associated (IL-6 and TNF-α) and M2-associated (Arg-1 and TGF-β1) markers across treatment groups, as measured by ELISA. (d, e) Representative Western blot images showing the protein expression of IL-6, iNOS, TGF-β1, and Arg-1. (f) Flow cytometric analysis of pulmonary M1 (CD86^+^) and M2 (CD206^+^) macrophage subsets. (g) Flow cytometric analysis of neutrophils (Ly6G^+^CD11b^+^) and mononuclear phagocytes (CD45^+^CD14^+^) in lung tissue. Data are presented as mean ± SD (n ≥ 3). *P < 0.05, **P < 0.01, ***P < 0.001.

Given that M2 macrophage activation and TGF-β1 signaling are central drivers of fibrogenesis, we next examined the pro-fibrotic M2 markers Arg-1 and transforming growth factor-β1 (TGF-β1). Serum ELISA (Fig. 6c) showed that BLM challenge significantly elevated Arg-1 and TGF-β1 levels, whereas nanomedicine treatment effectively reversed these increases. Western blot analysis of lung tissues collected at the treatment endpoint (Fig. 6d, e and Fig. S11) further demonstrated that both PFD- and MPS-loaded nanoformulations downregulated the M1 markers IL-6 and iNOS, as well as the M2 markers Arg-1 and TGF-β1, confirming their dual anti-inflammatory and anti-fibrotic activities at the protein level. Among all formulations, CMPNPs achieved the greatest suppression of these markers, which can be attributed to their superior tissue penetration and enhanced drug accumulation within fibrotic lesions.

Flow cytometric analysis of lung immune cell populations (Fig. 6f) revealed the cellular basis of these immunomodulatory effects. Consistent with the in vitro polarization results, both M1 (CD86^+^) and M2 (CD206^+^) macrophages were significantly enriched in the lungs at 21 days post-BLM, reflecting sustained inflammatory activation and pro-fibrotic remodeling, respectively. Treatment with the drug-loaded nanomedicines markedly reduced both subsets, with the strongest effect in the CMPNPs group. CMPNPs also restored the CD86/CD206 (M1/M2) ratio toward homeostatic levels (Fig. S12), indicating a genuine rebalance of macrophage polarization rather than a uniform change in macrophage abundance. The single-drug formulations revealed complementary roles for the two payloads. MPS (in MNPs) preferentially suppressed the pro-inflammatory M1 subset, whereas PFD (in PNPs) predominantly limited the pro-fibrotic M2 population. Such complementary M1/M2 regulation may underlie the superior efficacy of the co-loaded CMPNPs. Beyond macrophages, BLM-challenged mice exhibited a marked expansion of CD11b^+^Ly6G^+^ neutrophils (Fig. 6g), which damage the alveolar epithelium, activate fibroblasts, and promote collagen deposition through the release of elastase, reactive oxygen species, and neutrophil extracellular traps (NETs). Nanomedicine treatment attenuated this neutrophil accumulation, with CMPNPs again the most effective. BLM challenge likewise induced the accumulation of CD45^+^CD14^+^ mononuclear phagocytes in lung tissue. These cells can differentiate into fibroblasts and directly synthesize collagen, thereby promoting IPF progression [55], in part by amplifying inflammation through the M-CSF/CCL2 axis [56]. Treatment with CMPNPs significantly suppressed this population as well.

The simultaneous suppression of M1 and M2 markers arises from several complementary mechanisms. First, MPS inhibits NF-κB signaling and thereby restrains M1-driven inflammation; at the same time, it markedly reduced pulmonary TGF-β1 (Fig. 6c–e), the principal cytokine driving pro-fibrotic M2 polarization in BLM-injured lungs, so depletion of this signal shrinks the TGF-β1–dependent M2 subset [57]. This pathological M2 population differs from the reparative M2 phenotype induced by IL-4 in vitro, which accounts for the divergent glucocorticoid effects reported across experimental settings. Second, PFD reinforces M2 suppression by blocking TGF-β1/Smad signaling [58], [59], and by alleviating matrix deposition it further weakens stiffness-mediated M2 activation. Third, collagenase does not act on macrophages directly; by degrading the accumulated collagen, it softens the fibrotic matrix, removes the mechanical cue favoring M2 polarization, and limits ECM-derived damage-associated molecular patterns (DAMPs) that sustain M1 activation [60]. Finally, because fibrosis and macrophage polarization reinforce each other in a positive feedback loop, attenuating fibrosis itself further dampens both phenotypes. CMPNPs thus act at multiple nodes of this loop to restore pulmonary immune homeostasis. In light of these complementary mechanisms, we believe that MPS and PFD act synergistically in the treatment of IPF rather than producing merely additive effects. Nevertheless, quantitative pharmacological indices such as the combination index will be determined in our future work to rigorously define this synergy.

### 3.7 Superior anti-fibrotic efficacy of inhalation versus intravenous delivery

Inhaled drug delivery offers direct lung targeting and high local bioavailability, providing a strategic advantage for treating pulmonary diseases [61]. To directly evaluate whether the administration route translates into differential therapeutic exposure, we compared the biodistribution of CMPNPs following intratracheal inhalation versus intravenous (i.v.) injection in BLM-induced IPF mice using fluorescence imaging. After intratracheal administration, the nanoparticles were almost entirely retained in the lungs, with negligible signal in off-target organs (Fig. 7a). In contrast, i.v.-administered CMPNPs accumulated mainly in the liver, followed by the lungs. Notably, at 24 h post-administration, the inhalation group still exhibited strong pulmonary fluorescence, whereas the signal in the i.v. group had largely faded, indicating rapid systemic clearance and markedly lower pulmonary retention (Fig. 7b). These findings demonstrate that inhalation maximizes lung-specific bioavailability and prolongs local drug retention while minimizing systemic exposure and potential off-target effects, providing a pharmacokinetic rationale for the superior efficacy of inhaled CMPNPs.

**Fig. 7:**
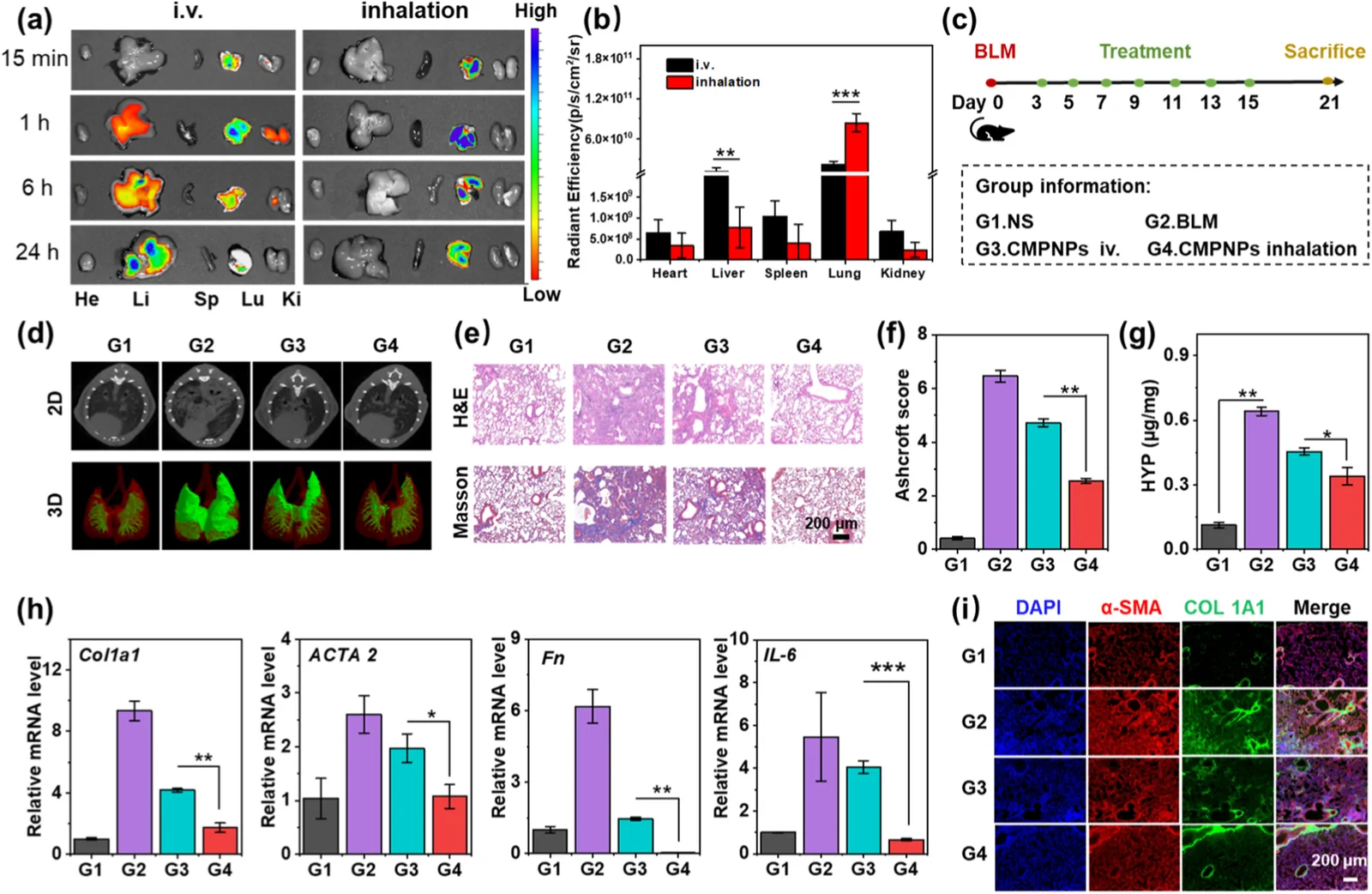
Comparison of the anti-fibrotic efficacy of CMPNPs delivered by inhalation versus intravenous injection. (a) Ex vivo fluorescence imaging of the biodistribution of Cy5.5-labeled CMPNPs in major organs at 15 min, 1, 6, and 24 h after intravenous (i.v.) injection or inhalation. (b) Quantitative analysis of fluorescence intensity of CMPNPs in major tissues at 24 h post-administration (i.v. vs. inhalation). (c) Schematic illustration of the experimental timeline and group allocation. (d) Representative cross-sectional (transverse) CT images of the lungs and 3D reconstructed CT images for each treatment group on day 21 post-BLM induction (burgundy: healthy tissue < 600 HU; green: fibrotic tissue > 600 HU). (e) Representative images of H&E and Masson's trichrome staining of lung tissue from each treatment group. (f) Ashcroft score analysis of Masson pathological staining of mouse lung. (g) HYP content in lung tissue across treatment groups. (h) Relative mRNA expression of fibrotic (Col1a1, ACTA2, Fn) and inflammatory (IL-6) markers in lung tissue across treatment groups, normalized to Gapdh. (i) Representative immunofluorescence images depicting co-localization of α-SMA (red) and COL1A1 (green) in lung tissue sections from each treatment group. Data are expressed as mean ± SD (n ≥ 3); *P < 0.05, **P < 0.01.

We then compared the efficacy of CMPNPs administered via intravenous (i.v.) injection versus inhalation in the BLM-induced model (Fig. 7c). Horizontal CT scans and corresponding 3D reconstructed images revealed that inhalation almost completely resolved fibrotic lesions, as evidenced by a marked reduction in high-density shadows in lung tissue. In contrast, i.v. delivery achieved only partial structural improvement (Fig. 7d). Histological analyses using H&E and Masson's trichrome staining confirmed that inhalation effectively reversed alveolar collapse and collagen deposition, whereas i.v. treatment provided only moderate amelioration (Fig. 7e). Semi-quantitative analysis of Masson's trichrome staining based on the Ashcroft scoring system yielded scores of 2.57 and 4.72 for the inhalation and i.v. groups, respectively (Fig. 7f), confirming a milder degree of pulmonary fibrosis with inhaled delivery. Consistently, as displayed in Fig. 7g, pulmonary HYP content was significantly lower in the inhalation group than in the i.v. group.

The superior structural outcomes with inhalation were consistent with molecular changes. Inhalation induced a stronger downregulation of key fibrotic genes (Col1a1, ACTA2, Fn, and IL-6) compared with i.v. injection (Fig. 7h). Immunofluorescence imaging showed markedly reduced expression of α-SMA and COL1A1 in lung tissue after inhalation relative to i.v. injection (Fig. 7i). Moreover, immunohistochemistry revealed a more pronounced reduction in the pulmonary expression of pro-inflammatory cytokines (IL-6, TNF-α, IL-1β) following inhalation versus systemic administration (Fig. S13). Collectively, these results demonstrate that inhalation enhances lung-targeted delivery of CMPNPs, leading to better preservation of pulmonary architecture and more comprehensive suppression of fibrotic pathways compared with intravenous injection.

### 3.8 In vivo safety evaluation

Beyond efficacy, a preliminary biosafety assessment of CMPNPs was conducted following repeated intratracheal administration. Histological analysis of major organs (heart, liver, spleen, and kidneys) on day 21 revealed no treatment-related pathological changes compared with healthy controls (Fig. S14a), and serum biochemical markers of hepatic injury (ALT, AST) and renal function (BUN, CRE) remained within normal physiological ranges (Fig. S14b), indicating no overt acute toxicity. Nevertheless, as this assessment was confined to a single time point, the risk of delayed immune-related adverse reactions against the exogenous collagenase upon repeated dosing cannot be fully excluded. This risk is expected to be low because inhalation delivery confines the enzyme largely to the lung tissue and minimizes systemic exposure. This expectation is supported by clinical experience with repeated collagenase therapy, in which anti-drug antibodies developed in most patients but were associated with neither hypersensitivity nor increased adverse events [62], [63]. Even so, long-term immunogenicity monitoring over extended observation windows will be essential for the future translational development of CMPNPs.

## 4 Conclusions

In summary, we have developed an inhalable, collagenase-modified liposomal nanoplatform (CMPNPs) co-encapsulating methylprednisolone (MPS) and pirfenidone (PFD) to address multiple pathological barriers in IPF. The surface-anchored collagenase enables degradation of the dense collagen-rich ECM, thereby facilitating nanoparticle penetration and drug delivery to fibrotic lesions. Concurrently, the dual drug payload modulates macrophage polarization away from a pro-fibrotic M2 phenotype and directly suppresses fibroblast activation. In a BLM-induced murine model, intratracheal administration of CMPNPs achieved superior anti-fibrotic and anti-inflammatory efficacy compared with non-enzymatic or single-drug formulations, leading to markedly reduced collagen deposition, restored alveolar architecture, and improved survival rate. This multi-targeted, barrier-penetrating strategy overcomes the poor drug accumulation and limited efficacy against complex pathology that limit current IPF therapies, and our findings support the translational potential of CMPNPs as a promising inhalable therapeutic for progressive pulmonary fibrosis.

## CRediT authorship contribution statement

**Yuyao Sun:** Writing – original draft, Methodology, Formal analysis. **Chenggong Yu:** Writing – review & editing, Supervision, Funding acquisition, Conceptualization. **Chaochao Zhang:** Methodology. **Ying Li:** Resources, Methodology, Formal analysis. **Wenqing Zhang:** Methodology. **Bowei Li:** Formal analysis. **Mengli Song:** Methodology. **Lin Wang:** Supervision. **Bowen Li:** Writing – review & editing, Supervision, Project administration. **Qingwen Xu:** Writing – review & editing, Validation, Supervision, Funding acquisition, Conceptualization.

## Declaration of competing interests

The authors declare that they have no known competing financial interests or personal relationships that could have appeared to influence the work reported in this paper.

## Data Availability

Data will be made available on request.
