# Supplementary material for: A multi-targeted, barrier-penetrating inhalable nanoplatform for simultaneous immunomodulation and antifibrotic therapy in idiopathic pulmonary fibrosis
Source: Mater Today Bio. 2026 Nov 6;41:103805. doi: 10.1016/j.mtbio.2026.103805 (PMC13769518; doi:10.1016/j.mtbio.2026.103805)
Supplement: Supplementary file 1 [file mmc1.docx]

**Supporting Information**

**A multi-targeted, barrier-penetrating inhalable nanoplatform for simultaneous immunomodulation and antifibrotic therapy in idiopathic pulmonary fibrosis**

Yuyao Sun^a,b,1^, Chenggong Yu^b,d,1,^*, Chaochao Zhang^b,d,1^, Ying Li^b,d^, Wenqing Zhang^b,d^, Bowei Li^b,d^, Mengli Song^b,d^, Lin Wang^a,*^, Bowen Li^c,^*, Qingwen Xu^a,b,^*

^a^Department of Pharmacy, the First Affiliated Hospital of Wannan Medical University (Yijishan Hospital of Wannan Medical University), Wuhu 241001, China

^b^School of Pharmacy, Wannan Medical University, Wuhu 241002, China

^c^School of Pharmacy, Anhui Medical University, Hefei 230032, P. R. China

^d^Anhui Innovative Center for Drug Basic Research of Metabolic Diseases, Wannan Medical University, Wuhu 241002, China

^1^These authors contributed equally to this work.

*Corresponding authors:

Department of Pharmacy, the First Affiliated Hospital of Wannan Medical University (Yijishan Hospital of Wannan Medical University), Wuhu 241001, China

Email: [qingwenxu@zju.edu.cn](mailto:qingwenxu@zju.edu.cn) (Q. Xu); [1033464919@qq.com](mailto:1033464919@qq.com) (L. Wang)

School of Pharmacy, Anhui Medical University, Hefei 230032, P. R. China

Email: bowen_li@ahmu.edu.cn (B. Li)

School of Pharmacy, Wannan Medical University, Wuhu 241002, China

Email: cgyu2024@wnmc.edu.cn (C. Yu).

**Fig. S1**. Infrared (IR) spectra of ColE, DSPE-PEG, and DSPE-PEG-ColE.

**Fig. S2.** UV-vis absorption spectra of DSPE-PEG and DSPE-PEG-ColE.

**Fig. S3**. Relative collagenase activity of ColE and DSPE-PEG-ColE.

**Fig. S4**. UV-vis absorption spectra of PFD and MPS.

**Fig. S5.** Hydrodynamic diameters of the nanoparticles monitored over 10 days in PBS, 10% fetal bovine serum (FBS), and deionized water (H₂O).

**Fig. S6.** (a) UV–vis absorption spectra of CMPNPs before and after nebulization. (b–d) Hydrodynamic size (b), polydispersity index (PDI) (c), and zeta potential (d) of various nanoformulations before and after nebulization.

**Fig. S7**. Confocal laser scanning microscopy (CLSM) images of MLg cells after culture with CMPNPs.

**Fig. S8.** Relative mRNA expression of fibrotic markers (Col1a1, Acta2, Fn, TGF-β1) in lung tissue across treatment groups, normalized to Gapdh.

**Fig. S9**. Representative immunofluorescence images showing the expression and co‑localization of α‑SMA and COL1A1 in mouse lung tissues.

**Fig. S10.** Relative mRNA expression levels of M1 (IL-6, TNF-α, IL-1β, and iNOS) and M2 (Arg-1) markers in lung tissue across treatment groups, normalized to Gapdh.

**Fig. S11.** Quantification of IL-6, iNOS, TGF-β1, and Arg-1 protein expression in lung tissue across different treatment groups, normalized to Gapdh.

**Fig. S12.** CD86/CD206 ratio (M1/M2) quantified by flow cytometry in different treatment groups.

**Fig. S13.** Representative immunohistochemical images showing the expression of IL‑6, TNF‑α, and IL‑1β in lung tissue sections from the indicated experimental groups.

**Fig. S14**. Preliminary in vivo safety assessment of CMPNPs. (a) Representative H&E-stained sections of the heart, liver, spleen, and kidney from healthy control mice and CMPNPs-treated IPF model mice at day 21. (b) Plasma levels of blood urea nitrogen (BUN), creatinine (CRE), alanine aminotransferase (ALT), and aspartate aminotransferase (AST) in the indicated groups at the study endpoint.

**Table S1** Primer sequences used in RT-qPCR experiment

| Gene | Sense (5'-3') | Antisense (5'-3') |
| --- | --- | --- |
| *Acta2* | TGCTGACAGAGGCACCACTGAA | CAGTTGTACGTCCAGAGGCATG |
| *Fn1* | CCCTATCTCTGATACCGTTGTCC | TGCCGCAACTACTGTGATTCGG |
| *Col1a1* | CCTCAGGGTATTGCTGGACAAC | CAGAAGGACCTTGTTTGCCAGG |
| *Gapdh* | CATCACTGCCACCCAGAAGACTG | ATGCCAGTGAGCTTCCCGTTCG |
| *TGF-β1* | CGAAGCGGACTACTATGCTAAAGG | TGGTTTTCTCATAGATGGCGTG |
| *IL-6* | TAGTCCTTCCTACCCCAATTTCC | TTGGTCCTTAGCCACTCCTTC |
| *TNF-α* | CCCTCACACTCACAAACCAC | ATAGCAAATCGGCTGACGGT |
| *IL-1β* | TGCCACCTTTTGACAGTGATG | TGATGTGCTGCTGCGAGATT |
| *IL-10* | GCAACCTGCCTAACATGCTTC | TTGGCAACCCAGGTAACCCTTA |
| *CD206* | TGGAACCACCACTGACTACG | TCTCGCTTCCCTCAAAGTGC |
| *CD86* | TGTTTCCGTGGAGACGCAAG | TTGAGCCTTTGTAAATGGGCA |
| *Arg-1* | CAAGACAGGGCTCCTTTCAG | GTAGTCAGTCCCTGGCTTATGG |
| *iNOS* | GTTCTCAGCCCAACAATACAAGA | GTGGACGGGTCGATGTCAC |
